# A perspective on the future of heart failure research

**DOI:** 10.1016/j.jmccpl.2026.100835

**Published:** 2026-02-07

**Authors:** Alyssa C. Vadovsky, Eunji Jeong, Sandeep Banga, Jason N. Bazil

**Affiliations:** a Department of Physiology, Michigan State University, East Lansing, MI, United States of America; b Central Michigan University, College of Medicine, Mount Pleasant, MI, United States of America; c Department of Cardiology, Sparrow Hospital, Michigan State University, Lansing, MI, United States of America; d Department of Biomedical Engineering, Michigan State University, East Lansing, MI, United States of America

**Keywords:** Heart failure, Bioenergetic abnormality, Mitochondrial dysfunction, Calcium phosphate, Oxidative stress

## Abstract

Heart failure is chronic condition that is diagnosed when the heart is no longer able to pump enough blood to meet the physiological needs of the body. It is a progressive condition and typically manifests early on as exercise intolerance due to insufficient cardiac output before developing into the end stage disease. Nearly 7 million U.S. adults currently live with some form of heart failure, roughly accounting for 14% of all deaths. Various etiologies of heart failure have been identified which stem from a constellation of perfusion, pressure, morphological, electrical, and/or metabolic origins. An underlying issue for nearly all forms of heart failure is what is known as bioenergetic abnormalities. Although mitochondrial dysfunction is widely implicated, the precise molecular mechanisms underlying bioenergetic abnormalities remain incompletely understood. In this commentary, we describe the most prevalent heart failure types and offer a possible mechanism capable of explaining these bioenergetic abnormalities. We explore a novel hypothesis that links oxidative stress caused by intrinsic and extrinsic factors, which leads to depressed oxidative capacities induced by the presence of mitochondrial calcium phosphate granules. This hypothesis is supported by evidence previously reported in literature and may offer a new etiology of heart failure. We conclude by outlining a bold strategy to advance heart failure research through innovative approaches and unexplored domains.

## Graphical abstract

**Figure:**
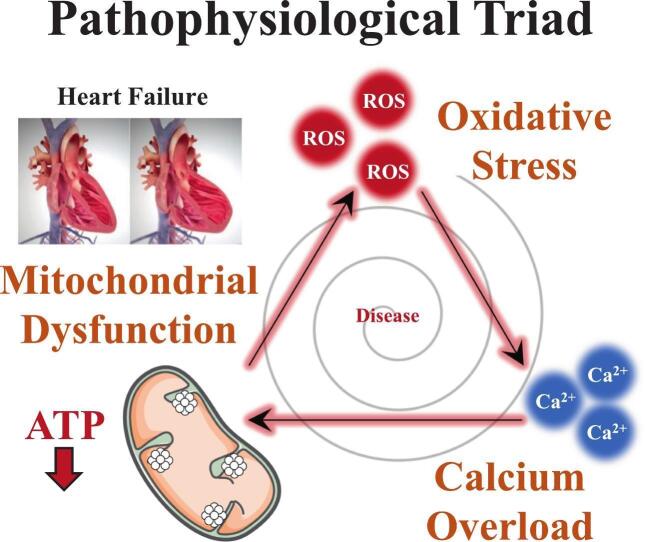

## 1 Introduction

Heart failure is described as a complex, multifactorial clinical syndrome defined by the heart's inability to maintain adequate output to meet systemic metabolic demands. Clinically, heart failure is categorized as heart failure with reduced ejection fraction (HFrEF) or heart failure with preserved ejection fraction (HFpEF), each with distinct hemodynamic profiles but converging at the cellular and metabolic level through overlapping pathologies [1], [2], [3], [4]. In many cases, oxidative stress, calcium overload, and mitochondrial dysfunction precipitate impaired ATP production and contractile failure. The causal chain that connects these three pathologies is typically in the order of calcium overload, oxidative stress, and then mitochondrial dysfunction; however, we will introduce series of arguments that explain how the start of metabolic impairment can be caused by oxidative stress which triggers calcium overload and precipitates mitochondrial dysfunction.

In general, oxidative stress, calcium overload, and mitochondrial dysfunction contribute to maladaptive cardiac remodeling, myocyte apoptosis, and ultimately, the clinical syndrome of heart failure [5], [6], [7], [8], [9]. The role of environmental factors and exposure to harmful chemicals has recently been highlighted as a possible contributor to heart disease, but research on this link is limited. We first discuss the main types of heart failure and their connection to mitochondrial dysfunction as a preamble. Then, we discuss a new mechanism of persistent, chronic oxidative stress which precipitates mitochondrial dysfunction through the formation of mitochondrial calcium phosphate granules. We then discuss how certain xenochemicals may be putting us at higher risks of cardiomyopathies or other diseases by creating pro-oxidant environments within living tissues. Next, we summarize how changes in bioenergetics and the impact of exogenous oxidants are worthy avenues of study to further pinpoint the causal elements of heart failure progression. In the end, we propose an ambitious plan to push heart failure research into the next frontier.

## 2 Ischemic heart disease and hypertension

Among the most prominent contributors to heart failure pathogenesis are ischemic heart disease (IHD) and hypertension that initiate cellular injury through perpetuating a vicious, self-amplifying cycle of redox imbalance and mitochondrial damage [10]. Oxidative stress—defined as an excess of reactive oxygen species (ROS) relative to antioxidant defenses—serves as a central mediator in both conditions. In IHD, oxidative stress accelerates endothelial dysfunction, atherosclerosis, and cardiomyocyte ischemia. In hypertension, oxidative stress drives vascular remodeling, calcium dysregulation, and concentric left ventricular hypertrophy [11], [12]. Aging accelerates oxidative stress and low-grade, chronic inflammation is suspected to play a major role in the development of heart failure; however, key details are still missing [13], [14]. Whether through pressure overload, volume overload, or metabolic insult, these upstream stressors ultimately converge on mitochondrial pathways that underlie both forms of heart failure. Thus, heart failure is not solely an impairment to the pumping mechanisms, but a state of profound bioenergetic dysfunction that causes the health of the organ to decline.

## 3 The role of mitochondrial dysfunction in HFrEF

Heart failure with reduced ejection fraction is defined as a clinical syndrome characterized by symptoms and/or signs of heart failure with a left ventricular ejection fraction (LVEF) of ≤40%. The hallmark of HFrEF is systolic dysfunction, where the left ventricle is unable to generate sufficient contractile force to maintain adequate cardiac output, leading to reduced stroke volume and ejection fraction. This impaired contractility is often accompanied by progressive ventricular dilatation and adverse remodeling, which further compromise systolic performance [15], [16], [17]. Volume overload is a central feature of HFrEF, which leads to increased preload, elevated ventricular filling pressures, and clinical manifestations such as pulmonary and peripheral edema. The resulting congestion is a major driver of symptoms (e.g., dyspnea, orthopnea, and exercise intolerance) and hospitalizations in HFrEF [18]. Three common underlying causes and precipitating events of HFrEF are atherosclerosis, myocardial infarction, and dilated cardiomyopathy.

Atherosclerosis is a central driver of ischemic heart disease and a major cause of HFrEF. It imposes significant metabolic stress on the myocardium through lipid peroxidation, iron-catalyzed ROS generation, and ferroptosis, all of which are supported by recent literature as contributors to plaque instability and endothelial apoptosis [19], [20]. Iron accumulation within plaques catalyzes lipid peroxidation, promoting ferroptosis and plaque progression [19]. Excessive ROS generation, from both mitochondrial and non-mitochondrial sources, further damages endothelial cells and promotes apoptosis, inflammation, and vascular dysfunction [20], [21]. Endothelial dysfunction in atherosclerosis reduces nitric oxide (NO) bioavailability, leading to vasoconstriction, inflammation, and impaired oxygen delivery to mitochondria [22]. This compromises the ATP production efficiency of fatty acid β-oxidation, and the heart relies more on carbohydrates for ATP [6], [10]. While fuel preference shifts, sympathetic activation increases circulating free fatty acids and myocardial lipid uptake as temporary compensation. Eventually, metabolic inflexibility and lipid accumulation follow [6], [23]. Residual or dysregulated fatty acid oxidation under hypoxic conditions is associated with oxidative stress that results in mitochondrial dysfunction [10], [21], [24]. This cascade of metabolic and redox disturbances is a key observation linking atherosclerosis, ischemic injury, and the development of HFrEF to bioenergetic impairments.

Myocardial infarction is often the culmination of atherosclerosis that leads to necrosis, inflammation, and maladaptive ventricular remodeling. Upon reperfusion following a myocardial infarction, a burst of ROS originating from mitochondrial complex I contributes to ischemia–reperfusion injury [25]. This ROS production is primarily driven by the oxidation of succinate accumulated during ischemia [26], [27]. Complex II oxidizes any excess succinate to generate an extremely reduced quinone pool and membrane potential hyperpolarization which leads to reverse electron transport through complex I [28]. This acute ROS surge damages proteins, lipids, and mitochondrial DNA and promotes posttranslational modifications that disrupt electron flow and ATP synthesis. Mitochondrial calcium overload also occurs due to dysregulated calcium handling, which, together with ROS, induces mitochondrial membrane depolarization and mitochondrial dysfunction. Loss of membrane potential halts, and in some cases reverses, ATP production, which depletes critical metabolites and activates necrotic and apoptotic cell death pathways [29], [30], [31], [32]. In the chronic phase of HFrEF, these mitochondrial insults persist, leading to sustained energetic dysfunction. Functional imaging studies, such as ^31^P nuclear magnetic resonance (NMR) spectroscopy, consistently demonstrate that patients with HFrEF have reduced myocardial phosphocreatine-to-adenosine triphosphate (PCr/ATP) ratios at rest, reflecting diminished energetic capacities [33]. Lower PCr/ATP ratios are strongly associated with worse clinical outcomes, supporting the concept that HFrEF is a state of bioenergetic dysfunction. This energetic deficit is driven by persistent mitochondrial dysfunction, ongoing ROS production, and impaired substrate utilization, all of which perpetuate contractile failure and adverse remodeling [34].

Atherosclerosis and myocardial infarctions are common ischemic conditions associated with IHD; however, common idiopathic or non-ischemic causes of HFrEF include dilated cardiomyopathy (DCM). This condition is defined by ventricular chamber enlargement and systolic dysfunction. Etiologies include genetic mutations, toxins, infections, and metabolic derangements. Histologically, DCM is marked by interstitial fibrosis, cytoskeletal disruption, and myofibril loss—hallmarks of energetic collapse and contractile failure [35], [36], [37]. Mitochondrial dysfunction is a central feature in DCM pathogenesis. It disrupts cardiac energy homeostasis and contributes to the transition from compensatory adaptation to progressive heart failure [35], [37], [38], [39]. The American Heart Association highlights that both nuclear and mitochondrial DNA mutations can directly cause or exacerbate cardiomyopathy, and that age-related accumulation of mitochondrial DNA mutations further contributes to disease progression [40]. Proteomic and translational studies in human DCM tissue confirm widespread alterations in mitochondrial protein expression, substrate utilization, and antioxidant defenses, with upregulation of stress response proteins such as PRDX3 correlating with disease severity [38], [41]. Environmental and metabolic stressors—such as chronic neurohormonal activation, oxidative stress, and metabolic inflexibility—drive excessive production of ROS, leading to mitochondrial DNA damage, impaired electron transport system function, and dysregulation of calcium homeostasis [42], [43]. These processes result in impaired ATP production, energy starvation, and activation of cell death pathways, promoting myocyte loss and adverse ventricular remodeling [44], [45]. Mitochondrial cristae junction dysregulation and defective cardiolipin remodeling further compromise oxidative phosphorylation and contribute to the accumulation of dysfunctional mitochondria [46], [47], [48].

## 4 The role of mitochondrial dysfunction in HFpEF

Heart failure with preserved ejection fraction, defined by a preserved LVEF of ≥50%, accounts for approximately half of all heart failure cases and is increasingly prevalent, particularly among older adults with comorbidities such as hypertension, obesity, and diabetes. It is also the primary form of heart failure in women [49]. The pathophysiology of HFpEF is distinct from HFrEF, with the central feature being diastolic dysfunction due to increased left ventricular (LV) stiffness and impaired relaxation, often resulting from chronic pressure overload. This leads to elevated LV filling pressures, left atrial enlargement, pulmonary hypertension, and right ventricular dysfunction, despite a preserved ejection fraction [50], [51], [52]. Chronic, low-grade, systemic inflammation is linked to many diseases including HFpEF, and ROS are suspected to be a major underlying cause [53], [54]. The two common pathologies associated with HFpEF are hypertension and aortic stenosis.

Hypertension results in pressure overload that induces concentric LV hypertrophy and impairs ventricular compliance and diastolic filling. Molecular mechanisms include increased interstitial fibrosis, alterations in titin phosphorylation, and systemic inflammation, all contributing to the stiffened myocardium and abnormal diastolic function. These changes result in symptoms of congestion and exercise intolerance, even in the absence of significant systolic dysfunction [51], [55]. Neurohormonal activation leads to increased circulating catecholamines and promotes renal sodium retention [56]. These processes facilitate excessive calcium influx into both vascular smooth muscle cells and cardiomyocytes, resulting in intracellular calcium overload [57]. Dysregulated calcium handling impairs excitation–contraction coupling and diastolic relaxation, further contributing to myocardial stiffness and diastolic dysfunction [58], [59]. Calcium overload within mitochondria disrupts oxidative phosphorylation, impairs ATP production, and leads to excessive ROS accumulation. Dysfunctional complex I of the electron transport chain is a key source of mitochondrial ROS, which exacerbates oxidative stress, promotes endothelial dysfunction, and increases vascular stiffness [60], [61]. These changes collectively impair myocardial energetics and contribute to the progression of HFpEF.

Aortic stenosis (AS), as a prototypical pressure-overload condition, contributes to HFpEF primarily through the development of concentric left ventricular hypertrophy and impaired ventricular relaxation. Chronic pressure overload from AS induces adaptive concentric hypertrophy, which increases myocardial wall thickness and stiffness, impairing diastolic filling and relaxation. The hypertrophied myocardium has increased oxygen demand due to greater muscle mass and elevated wall stress, while coronary perfusion is compromised, due to the hemodynamic changes [62]. This mismatch between oxygen supply and demand results in ischemia, which in turn promotes increases oxidative stress and mitochondrial dysfunction. ATP synthesis is then impaired, which is critical for active calcium reuptake into the sarcoplasmic reticulum via SERCA2a, leading to abnormal calcium cycling and elevated diastolic cytosolic calcium. The mechanisms in AS-induced HFpEF closely parallel those described in hypertension-induced HFpEF, including neurohormonal activation, renal sodium and calcium retention, sympathetic overactivity, and endothelial dysfunction, all of which converge on increased myocardial stiffness and impaired relaxation [7], [63]. Thus, pressure overload from AS and hypertension share final common pathways of oxidative stress, calcium overload, and mitochondrial dysfunction which results in impaired energetics and culminates in the HFpEF phenotype.

## 5 Convergence on mitochondrial pathology

Mitochondrial dysfunction is a central feature of heart failure pathogenesis, with oxidative stress playing a pivotal role in both HFrEF and HFpEF. In HFrEF, increased ROS levels lead to oxidative damage of proteins, lipids, and nucleic acids, contributing to contractile dysfunction, maladaptive remodeling, and cell death. Human and animal studies consistently demonstrate elevated markers of oxidative stress and impaired mitochondrial antioxidant defenses in failing myocardium [9], [35], [64]. In HFpEF, ROS dysregulation is also prominent, with evidence from both human tissue and animal models revealing redox imbalance and downstream effects on endothelial function and myocardial stiffness [8], [65]. Notably, while both HFrEF and HFpEF exhibit oxidative stress markers, the upstream triggers may differ, with neurohormonal activation predominating in HFrEF and systemic inflammation more prominent in HFpEF [1]. Moreover, HFpEF may be more susceptible to mitochondrial dysfunction than HFrEF due to differing fatty acid or glucose oxidation rates [66]. The main question is what causes this persistent oxidative stress signal? Pathological responses to chronic homeostatic disruptions, such as hypertension [11], [67], [68], [69], can generate persistent oxidative stress signals that propagate maladaptive feedback loops within cardiovascular and other organ systems. Moreover, dysfunctional mitochondria are themselves major sources of oxidative stress, but whether they are induced into a state of dysfunction by preceding upstream events is yet to be determined.

Mitochondrial calcium overload is believed to be a major contributor to heart failure progression. In HFrEF, chronic neurohormonal stimulation and impaired calcium handling lead to excessive mitochondrial calcium uptake, promoting mitochondrial permeability transition, loss of membrane potential, and cell death [9], [36], [70]. Animal models and human studies confirm that the mitochondrial permeability transition phenomenon is associated with the progression from compensated hypertrophy to decompensated failure. This calcium- and ROS-sensitive phenomenon triggers the collapse of membrane potential, impairment of ATP production, and release of pro-apoptotic factors, including cytochrome *c*, from the mitochondrial network [71], [72]. Sustained calcium overload compromises the mitochondrial network further and culminates in cardiomyocyte death. The RIPK3–CaMKII axis further amplifies this injury by driving necroptosis and inflammation in cardiac tissue, especially during ischemia-reperfusion episodes and chronic heart failure states [73]. In HFpEF, recent comparative studies reveal enhanced cytosolic calcium release and altered mitochondrial polarization, with evidence of impaired calcium cycling and increased susceptibility to permeability transition, though the precise molecular triggers may differ from HFrEF [1]. Both phenotypes ultimately converge on mitochondrial dysfunction and energetic failure.

## 6 The bioenergetic abnormality

In nearly all cases of heart failure, a bioenergetic abnormality is recognized as the key component of the underlying pathophysiology [33]; however, the casual origin(s) of this bioenergetic abnormality is currently unknown. At clinical and research levels, this phenomenon is detected with ^31^P NMR spectroscopy and reveals low PCr/ATP ratios in living tissue [74], [75], [76]. This is typically interpreted as being caused by a problem with the rate of mitochondrial ATP production [8], [77]. Two studies on heart failure in humans have investigated mitochondria purified from human heart tissue and both came up with a similar conclusion: isolated mitochondria from failing hearts are not statistically different from donor control hearts when it comes to carbohydrate-based and fatty-acid based oxidative capacity [78], [79]. In 2014, Cordero-Reyes et al. concluded that impaired substrate supply or reduced mitochondrial content might be the cause of the bioenergetic abnormality. Two years later, Holzem et al. showed data indicating the lipid droplets, perilipin protein, and lipoprotein phospholipase levels are altered in a way that may explain the bioenergetic abnormality, in addition to, lipotoxicity found in failing hearts [80], [81]. They also showed that mitochondrial content between failing, and donor hearts was the same based on citrate synthase activity, a mitochondrial biomarker. Nine years later, Latchman et al. published a study corroborating these ultrastructural and lipid droplet findings from Cordero-Reyes et al. and Holzem et al. [82]. Altogether, these observational studies are among many that point towards issues related to energy metabolism, but the mitochondria themselves are not a major cause of the observed metabolic dysfunction.

While mitochondria clearly play a role in heart failure pathogenesis, it is hard to link the bioenergetic abnormality to intrinsic differences in oxidative capacity. While current evidence suggests that fuel delivery may be a problem, the adage that the heart is an engine out of fuel [4] is becoming less convincing as new ideas centered on fuel delivery and utilization are being proposed [83]. Limited pyruvate supply and/or oxidation have been observed in both HFpEF and HFrEF [6], [84], [85], [86]; however, the underlying causes appear to differ. While human studies report a reduction in glycolytic flux in HFpEF [87], findings from a rat model attribute energetic dysfunction to an uncoupling of glycolysis from glucose oxidation rather than pathway limitations [88]. These discrepancies highlight the pitfalls of certain animal models, underscoring the need to critically reassess animal-based research which should guide our efforts to solve this metabolic puzzle.

Research efforts should be focused on identifying the bioenergetic abnormality (low PCr/ATP ratios) detected in failing hearts at rest [33]. Interestingly, lower mitochondrial pyruvate carrier protein levels have been associated with heart failure [85]; therefore, it is possible that low matrix levels of pyruvate may contribute to the bioenergetic abnormality. Moreover, a recent computational study revealed that a reduction in PDH flux can recapitulate some of the well-known characteristics of heart failure metabolism [89]. It's important to note that in isolated mitochondrial studies, pyruvate is often supplied in excess well above physiological levels which activates the complex by inhibiting its kinase inhibitor [90]. Thus, the prior functional studies using mitochondria isolated from failing and donor hearts [78], [79] were unable to detect alterations of in vivo PDH activity. These studies showed no difference in pyruvate-dependent oxidative capacity between failing and donor heart mitochondria, which argues against the mitochondrial pyruvate carrier being rate limiting and responsible for lower ATP production levels. In failing hearts, pyruvate oxidation may become limiting when cytosolic pyruvate is in its physiological concentration range and the mitochondrial pyruvate carrier levels are reduced. Unfortunately, mitochondrial pyruvate carrier protein levels were not quantified in the Holzem et al. or Cordero-Reyes et al. studies, so more research on this possibility is needed. How the mitochondrial pyruvate carrier hypothesis integrates with the concept of fatty acid oxidation fueling the majority of ATP produced in the heart [91] is another factor that requires explaining. Regardless, the limiting pyruvate oxidation hypothesis has merit and is worthy of further investigation.

## 7 A new emergent hypothesis

**Fig. 1:**
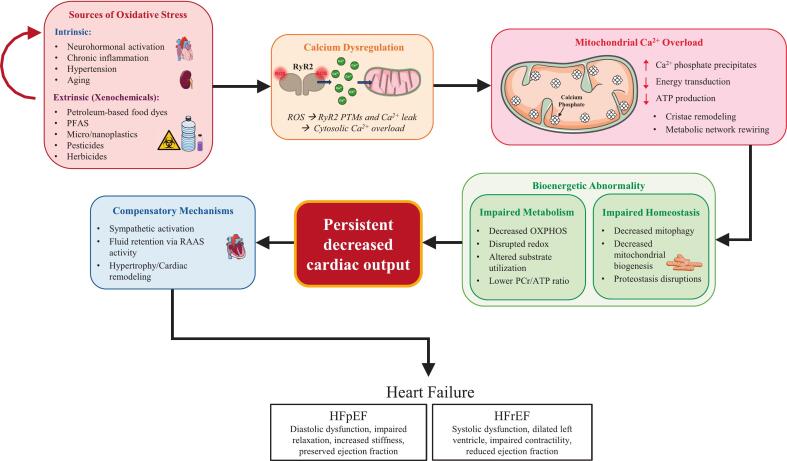
Sustained bioenergetic abnormalities drive heart failure through oxidative stress pathways that are triggered and exacerbated by environmental factors. In heart failure, a sustained reduction in cardiac output triggers compensatory mechanisms aimed at restoring homeostasis through diverse signaling pathways operating across spatial and temporal scales. Intrinsic factors such as aging, diet, lifestyle, and comorbidities contribute to oxidative stress that disrupts cellular metabolism. Extrinsic sources including xenochemicals from poor waste management and food processing, such as petroleum-based dyes, PFAS, microplastics, pesticides, and herbicides trigger and further exacerbate oxidative stress. Collectively, these stressors promote post-translational modifications (PTMs) of ryanodine receptors, impairing calcium handling and predisposing to mitochondrial calcium overload. Excess mitochondrial calcium leads to calcium phosphate precipitate formation, which alters cristae architecture and diminishes ATP production. This cascade underlies bioenergetic deficits observed clinically as reduced PCr/ATP ratios in heart failure patients. Impaired mitochondrial quality control, biogenesis, and proteostasis prevent restoration of metabolic homeostasis. Progressive decline in cardiac output drives phenotypic adaptations, including sympathetic activation, RAAS-mediated fluid retention, hypertrophic remodeling, and ultimately decompensation. Over time, these compensatory responses fail, culminating in pathologies such as HFpEF and HFrEF. The arrows do not necessarily correspond to casual connections, but instead show how these factors compound to promote disease progression.

Based on the studies discussed above, the data do not point to a specific causal mitochondrial defect but rather suggest involvement of extramitochondrial factors or other mechanisms that have thus far evaded detection. A novel hypothesis (Fig. 1) centered on calcium overload may be able to thread the needle and explain the bioenergetic abnormality in some cases of heart failure [92]. This hypothesis involves the concept of calcium phosphate precipitates disrupting cristae and lowering ATP output in a calcium-load dependent manner [93] without inducing the well-known mitochondrial permeability transition phenomenon [94]. In this calcium overloaded state, oxidative phosphorylation is impaired when NADH-linked substrates (e.g., pyruvate) are the fuel and not when succinate is used in the presence of rotenone, a complex I inhibitor that obstructs its Q-site [94]. The impairment is more severe when fats are the sole substrate when compared to pyruvate [94]. The presence of calcium phosphate precipitates in living cells was controversial until the advent of cryo-ET imaging which revealed their presence [95], [96], [97], [98], [99]. We can speculate a possible mechanism capable of explaining the bioenergetic abnormality which relies on the observation that calcium overload is associated with an increase in cristae junctional widths [100]. This increase in width would lead to redistribution of cytochrome *c* from the cristae lumen to the intermembrane space [101]. In theory, this redistribution can lead to reduced proton current [102] and would explain the lower ATP production rates and ultimately the bioenergetic abnormality seen in heart failure. We stress that this cristae junction hypothesis is very likely part of a set of changes that occur to explain why calcium overloaded mitochondria export ATP as a slower rate. The metabolic system that generates and delivers ATP to the cell contains many parts which can be heterogeneously impaired to result in the net effect of lower ATP production rates. It is entirely possible other mechanisms can explain the calcium-dependent drop in ATP production rates in addition to the cristae junction hypothesis. In other words, it's an effect size issue and a systems level perspective is required to elucidate this phenomenon. Nevertheless, the casual link between calcium overload and impaired ATP production is evident and supported by an abundance of data [93], [94], [103], [104], [105], [106], [107], [108], [109], [110].

## 8 The calcium overload problem

For the calcium phosphate hypothesis to gain credibility, it must withstand rigorous logical evaluation. First, if these granules are present in the failing heart, they will dissolve when mitochondria are extracted from the tissue, because these granules are labile in low to moderate calcium overload conditions [103]. When mitochondria are isolated, calcium-free buffers are used to prevent calcium overload during the isolation process [111]. Thus, the isolated mitochondria from human heart tissue in the Holzem et al. and Cordero-Reyes et al. studies would not contain any calcium phosphate granules and thus not be structurally and functionally compromised as in the in vitro studies [93], [100]. Second, can mitochondria become calcium overloaded in vivo and does that precipitate heart failure? Santulli et al. reported that a mouse model expressing a constitutively leaky RyR2 channel results in heart failure [112]. Expression of the leaky RyR2 channel led to higher mitochondrial calcium loads, lower mitochondrial ATP production rates, and decreased cristae density. Similar mitochondrial ultrastructural changes have also been observed in other studies centered on leaky RyR2 channels [113], [114]. Unfortunately, the limited number of EM images and their low publication resolution in studies of human heart failure [78], [82] or leaky RyR2 channel mutants [112], [113], [114] cannot be used to unambiguously identify the presence of granules, despite evidence of ultrastructural changes; however, older studies on ischemia/reperfusion injury have reported them [115], [116], [117], [118], [119]. Overall, these findings align with the calcium phosphate hypothesis described above. Thus, calcium phosphate granule formation could be driven by leaky RyR channels, leading to mitochondrial dysfunction and pathogenesis. In a separate but related study, the calcium phosphate hypothesis also explains the results reported by Garbincius et al. where they found a form of heart failure that appears to be caused by calcium overload but independent of the mitochondrial permeability transition phenomenon [120]. This hypothesis can also help explain to some degree the decrease in contractility associated with heart failure [121], [122]. As mitochondrial ATP output decreases, Pi will increase after a new metabolic steady state is achieved. An increase in Pi has been detected in failing hearts [123], [124]. And higher Pi levels have been linked to a reduction in force generation in cardiac tissue [125], [126], but the effect size on cardiac output has not yet been determined. While these human-based studies and results from animal models provide supporting evidence for the calcium phosphate hypothesis as an additional failing heart etiology, human studies using energy-dispersive x-ray spectroscopy or some other form of elemental analysis are needed to confirm it.

## 9 Housecleaning gone awry

But are mitochondria containing calcium phosphate granules present during the development of heart failure, which would help explain the bioenergetic abnormality observed at the clinical level? Under normal conditions, as energy production becomes limiting, pathways that promote the genesis of new mitochondria [127] and others ensure that nonfunctional or damaged mitochondria are cleared from the cell [128]. When calcium handling becomes dysregulated as seen in the development of both HFrEF and HFpEF, mitochondria overloaded with calcium would be detected by the cell and selectively removed via mitophagy [128], [129]; however, mitophagy is impaired in heart failure. In HFrEF, blunted mitophagy leads to accumulation of dysfunctional mitochondria, exacerbating oxidative stress and contractile dysfunction [9], [36]. In HFpEF, recent work in mouse models and human iPSC-derived cardiomyocytes demonstrates that mitophagy is not appropriately activated in response to metabolic stress, resulting in defective mitochondrial quality control and contributing to energetic deficits and disease progression [130]. This work showed that enhancing mitophagy through metabolic interventions can ameliorate HFpEF phenotypes in preclinical models. Another mechanism capable of addressing chronically calcium overloaded mitochondria is to increase biogenesis of new, healthy mitochondria. Unfortunately, mitochondrial biogenesis is reduced in failing rat and human hearts [131], [132], [133], [134], [135]. Altogether, these impaired processes will lead to the accumulation of dysfunctional mitochondria, which helps explain the bioenergetic abnormality observed in heart failure.

## 10 The impact of oxidative stress

How do mitochondria become loaded with calcium phosphate granules in the first place? RyR2 channels are sensitive to oxidative stress, which causes them to leak calcium into the cytosol [136]. In a pro-oxidative environment, the RyR2 channel can be oxidized at cysteines 1078 and 2991 which result in a pathological leaky RyR2 channel [137]. In addition, several phosphorylation sites have been identified as calcium leak inducers, and several labs are establishing the mechanisms responsible [138], [139]. The kinases and phosphatases regulating these sites are also subject to redox regulation [140], [141]. Thus, oxidative stress plays a foundational role in RyR2 regulation. Unsurprisingly, these leaky RyR2 channels are found in failing hearts [142] and are highly associated with right heart failure [143]. Like dysfunctional mitochondria, leaky RyR2 channels would be subject to cellular house cleaning programs; however, protein homeostasis is disrupted in both HFrEF and HFpEF. Increased protein post-translational modifications, misfolding, and aggregation are observed in animal models and human tissue [36], [144], [145], [146]. Hyperacetylation of mitochondrial and cytosolic proteins can impair enzymatic activity and mitochondrial function, while accumulation of damaged proteins further stresses the proteostasis network [147], [148], [149]. These changes are more pronounced in HFpEF, where inflammation-driven post-translational modifications exacerbate mitochondrial dysfunction [1]. Therefore, oxidative stress can induce mitochondrial calcium overload and precipitate heart failure, but what causes the initial oxidative stress signal that leads to leaky RyR2 channels and kick starts the pathological remodeling associated with heart failure? Whatever this signal is, it must be persistent and refractory to current therapeutic interventions.

## 11 Sources of oxidative stress

Oxidative stress is either produced endogenously from organelles and enzymes or exogenously from xenochemicals. In tandem, both mechanisms contribute to a prooxidative environment capable of driving heart failure progression. In a cardiac myocyte, the primary endogenous pathways include the mitochondrial enzymes [28], [150], NAPDH oxidases [151], [152], [153], xanthine oxidase [154], uncoupled nitric oxide synthase [155], and monamine oxidases [156]. Also, stimulation of *β*-adrenergic signaling, an early event in heart failure progression, leads to an acute increase in ROS production that subsides due to compensation in later stages [157]. The immune system is an additional endogenous source of oxidative stress in cardiovascular disease and heart failure [158]. The list of exogenous xenochemicals that contribute to oxidative stress is extensive and includes things such as *per*- and polyfluoroalkyl substances (PFAS), petroleum-based food coloring agents, herbicides, and pesticides [159], [160], [161], [162], [163], [164], [165], [166], [167]. Known mechanisms for some xenochemicals have been identified, but many other effects are correlative without precise mechanisms identified. Importantly, many petroleum-based food coloring agents are redox cyclers and can generate a pro-oxidative environment if they accumulate to high enough levels in living tissue. Xenochemicals can also indirectly cause oxidative stress via immune system modulation in a vicious cycle [168]. No known study has exhaustively explored xenochemicals and chronic diseases such as heart failure; however, it is likely that the exposure of xenochemicals corresponds to the increasing rates of heart failure and other chronic diseases.

## 12 Xenochemicals and the environment

The impact of xenochemicals on human health is gaining traction [169], [170], [171], [172], [173]. Due to pollution and ill waste management, detectable amounts of various heavy metals and chemicals have been reported [174]. Microplastics and nanoplastics are another cause for concern [175], [176]. They have recently become a topic of interest in cardiovascular health and research [177]. A study by Wei et al. found microplastics were embedded in the cardiac tissue of Wistar rats when they were exposed to polystyrene microplastics in their drinking water [178]. This exposure resulted in disrupted mitochondrial function, elevated oxidative stress, reduced antioxidant defenses, and pyroptosis. Furthermore, an observational human study found that in patients undergoing treatment for coronary artery disease, microplastics were detected in roughly 60% of the carotid plaque specimens analyzed. Among the compounds identified were polyethylene, polyvinyl chloride, and chlorine. Patients with detectible levels of these microplastics were at a higher risk of heart attack, stroke, and morbidity [179]. A study published in 2025 by Massie et al. also found nanoplastics in femoral artery plaques excised from patients with peripheral artery disease. Those with atherosclerosis had significantly more nanoplastic particles in comparison to the control group [180]. Research suggests that human intake of microplastics is through improper waste management, food production and packaging processes, and lifestyle behaviors, but this list is not exhaustive. Thus, there is an acute need for further exploration of how xenochemicals contribute to chronic disease progression and mitochondrial dysfunction through persistent oxidative stress. Overall, metabolic insults increase risk and occurrence of cardiomyopathies, which could be driven by exogenous oxidants through environmental exposure.

## 13 Alternative explanations of the bioenergetic abnormality

Mitochondrial uncoupling [181] and mitochondrial DNA damage inducing mitochondrial heteroplasmy [182] are other possible mechanisms capable of explaining the bioenergetic abnormality. Mitochondrial DNA damage is increasingly recognized as a contributor of heart failure considering that excessive oxidative stress can lead to mitochondrial DNA mutations and deletions, which impair mitochondrial function [36]. Leakage of mitochondrial DNA can also trigger inflammatory responses, further exacerbating oxidative stress and promoting maladaptive cardiac remodeling [35]. Both animal and human studies support a role for mitochondrial DNA damage in the progression of heart failure syndromes. Factors that induce mitochondrial uncoupling and DNA damage are an additional set of plausible heart failure etiologies that must be further explored. Many factors contribute to heart failure and other diseases at varying spatial and temporal levels of physiology, but it is evident that oxidative stress plays a fundamental role.

## 14 Conclusions and charge to the community

The occurrence of HFrEF and HFpEF elicit different presentations of symptoms but share mitochondrial dysfunction as a key pathophysiological feature. Emerging evidence implicates environmental factors, such as PFAS, additives in ultra-processed foods, and other environmental toxins, in disrupting mitochondrial physiology through mechanisms like oxidative stress, calcium dysregulation, and bioenergetic impairment, which contribute to heart failure progression. Although acute exposure to these chemicals may not have large detrimental health effects, they are not biologically inert and can accumulate in living tissue; therefore, quantifying circulating levels of these toxins and analyzing potential impacts on our physiology is critical. As environmental and dietary factors tend to cause changes chronically, longitudinal studies would be ideal for assessing the role of xenochemicals in the progression of metabolic diseases such as heart failure. Mitophagy, mitochondrial biogenesis, and protein synthesis are meant to repair damaged cell components, but in heart failure, these repair mechanisms are disrupted. This is presumably due to the presence of exogenous factors that act on these vital cellular pathways. There is a glaring need for future research to elucidate how environmental factors cause oxidative stress to better shape food and drug regulations and human exposure to harmful chemicals. Fortunately, institutional leaders are tackling this problem head on [183]; however, there is much more research that needs to be done in order to identify and remove the most pernicious xenochemicals using well-thought-out, humancentric, and cost-benefit analyses. In doing so, we can identify the root causes of the chronic health epidemic plaguing the human population and improve the quality of life for our children and generations to come.

## CRediT authorship contribution statement

**Alyssa C. Vadovsky:** Writing – review & editing, Writing – original draft, Data curation. **Eunji Jeong:** Writing – review & editing, Writing – original draft, Methodology, Formal analysis, Data curation. **Sandeep Banga:** Writing – review & editing, Supervision. **Jason N. Bazil:** Writing – review & editing, Writing – original draft, Validation, Supervision, Resources, Project administration, Methodology, Investigation, Funding acquisition, Formal analysis, Data curation, Conceptualization.

## Declaration of Generative AI and AI-assisted technologies in the writing process

During the preparation of this work the authors augmented search queries with Grok and Copilot to find relevant peer-reviewed studies.

## Funding sources

This work was funded in part by 10.13039/100000001NSF CAREER MCB 2237117.

## Declaration of competing interest

This work was funded in part by 10.13039/100000001NSF CAREER MCB 2237117. No funding from other third parties was used.

No financial interest or relationships within the last 3 years related to the subject matter.

No patents or copyrights relevant to the manuscript.

No additional relationships or interests that could be perceived as a conflict.
